# Cost-effectiveness of implant movement analysis in aseptic loosening after hip replacement: a health-economic model

**DOI:** 10.1186/s12962-023-00498-w

**Published:** 2023-11-20

**Authors:** Davide Lovera, Olof Sandberg, Maziar Mohaddes, Hanna Gyllensten

**Affiliations:** 1 Sahlgrenska Academy, University of Gothenburg, Gothenburg, Sweden; 2 Sectra, Linköping, Sweden; 3 Department of Orthopaedics, Institute of Clinical Sciences, Sahlgrenska Academy, University of Gothenburg, and Sahlgrenska University Hospital, Gothenburg, Sweden; 4 Institute of Health and Care Sciences, Sahlgrenska Academy, University of Gothenburg, P. O. Box 457, 405 30 Gothenburg, Sweden

**Keywords:** Cost-effectiveness analysis, Cost and cost analysis, Arthroplasty, Replacement, Hip/adverse effects, Reoperation, Tomography, X-Ray computed

## Abstract

**Objective:**

To investigate the cost-effectiveness of using Implant Movement Analysis (IMA) to follow up suspected aseptic loosening when the diagnosis after an initial X-ray is not conclusive, compared with a diagnostic pathway with X-ray follow-up.

**Methods:**

A health-economic model in the form of a decision tree was developed using quality-adjusted life years (QALY) from the literature, cost-per-patient data from a university hospital and the probabilities of different events from expert physicians’ opinions. The base case incremental cost-effectiveness ratio (ICER) was compared with established willingness-to-pay thresholds and sensitivity analyses were performed to account for assumptions and uncertainty.

**Results:**

The base case ICER indicated that the IMA pathway was cost effective (SEK 99,681, compared with the SEK 500,000 threshold). In the sensitivity analysis, the IMA pathway remained cost effective during most changes in parameters. ICERs above the threshold value occurred in cases where a larger or smaller proportion of people receive immediate surgery.

**Conclusion:**

A diagnostic pathway using IMA after an inconclusive X-ray for suspected aseptic loosening was cost effective compared with a pathway with X-ray follow-up.

**Supplementary Information:**

The online version contains supplementary material available at 10.1186/s12962-023-00498-w.

## Background

Total hip arthroplasty (THA) is a surgical procedure in which the damaged bone and cartilage in a hip is removed and substituted by a joint prosthesis, alleviating pain and/or restoring function [1, 2]. The use of this surgical procedure is increasing, partly due to population aging, resulting in higher numbers of hip fractures and osteoarthritis [3]. In Sweden, the number of hip fractures is expected to double by 2050, reaching approximately 30,000 [4].

Even though most hip surgeries are successful, severe adverse events can develop over time, some of which require revision surgery. Common adverse events after hip revision surgery include aseptic loosening, dislocation, periprosthetic fractures and infection [5]. In Sweden, aseptic loosening is the most common reason for revision [6]. In revision surgery, there is a higher risk of mortality and a smaller chance of success compared with a primary operation, highlighting the necessity of developing surgical pathways that minimize complications and avoid unnecessary revisions [7].

The standard diagnostic procedure for ruling out aseptic loosening is a plain X-ray. A plain X-ray is an informative, quick, and inexpensive method for diagnosing implant loosening. However, the sensitivity and specificity of an X-ray in diagnosing loosening are inadequate [8, 9]. In a proportion of patients where plain X-rays are inconclusive, further imaging modalities and a sequential follow-up X-ray is needed. As a result, there is a need for more accurate and cost-effective methods for diagnosing implant loosening, where technological advances are a possible aid in preventing unnecessary revision surgeries [10, 11]. SECTRA, a Swedish company active in medical technology, has developed a new diagnostic tool with increased precision called Implant Movement Analysis (IMA), using displacement CT with alternated rotation of the femur [12]. Two low-dose provocation-computed topography (CT) scans are performed, where the limb is first provoked in one direction for the first CT scan and then in the other direction for the second CT scan. The software is able to merge the two images and a specially trained radiologist analyzes the data to provide the surgeon with additional information that will help to diagnose whether and how the implant is loose and, ultimately, whether surgery should be recommended to the patient [9]. However, no economic evaluations have been published in relation to this novel diagnostic tool, resulting in a lack of knowledge to aid decision-making regarding its introduction in care pathways.

The overall aim of this project is to investigate the cost-effectiveness of using implant movement analysis (IMA) to follow up suspected aseptic loosening when the diagnosis after an initial X-ray is not conclusive, compared with a diagnostic pathway with X-ray follow-up.

## Methods

The study followed the ISPOR guidelines for good modeling practice [13] and was reported according to the Consolidated Health Economic Evaluation Reporting Standards (CHEERS) [14].

The methods section is structured as follows: first, a description of the model used, followed by a presentation of the way the model is populated based on literature and real-world evidence and, finally, a description of the way the model was tested through sensitivity analyses. The population target comprises patients who are suspected of having aseptic loosening after an X-ray and the considered time horizon is 2 years. Analyses of costs were conducted from a healthcare perspective, in line with an attempt to reduce the healthcare resources allocated to this diagnosis. Moreover, in a largely tax-based health system, like the Swedish system [15], the direct costs to individual patients for receiving healthcare will be limited and the population is older, thereby making the cost of lost productivity in the labor market largely redundant.

### Conceptual model: decision tree of diagnostic pathway with and without IMA

Modeling is a tool for authorities or experts to support decision-making and can be used to anticipate and predict the impact of specific healthcare interventions on a group of patients, individuals or society [16]. Decision trees are one type of decision-analysis model used for economic evaluations and in this study, they will be used to depict the process undergone after a suspected aseptic loosening. Decision trees are mainly valued for their simplicity and transparency and they are used as a mean of clarifying options of interest [17]. Decision trees start from an initial group of patients for whom a decision is made and then branch out based on the consequences of that decision. A cost and a health outcome that indicates the aggregated results for one person following that specific path, or branch, is attached to the end of each branch.

**Fig. 1:**
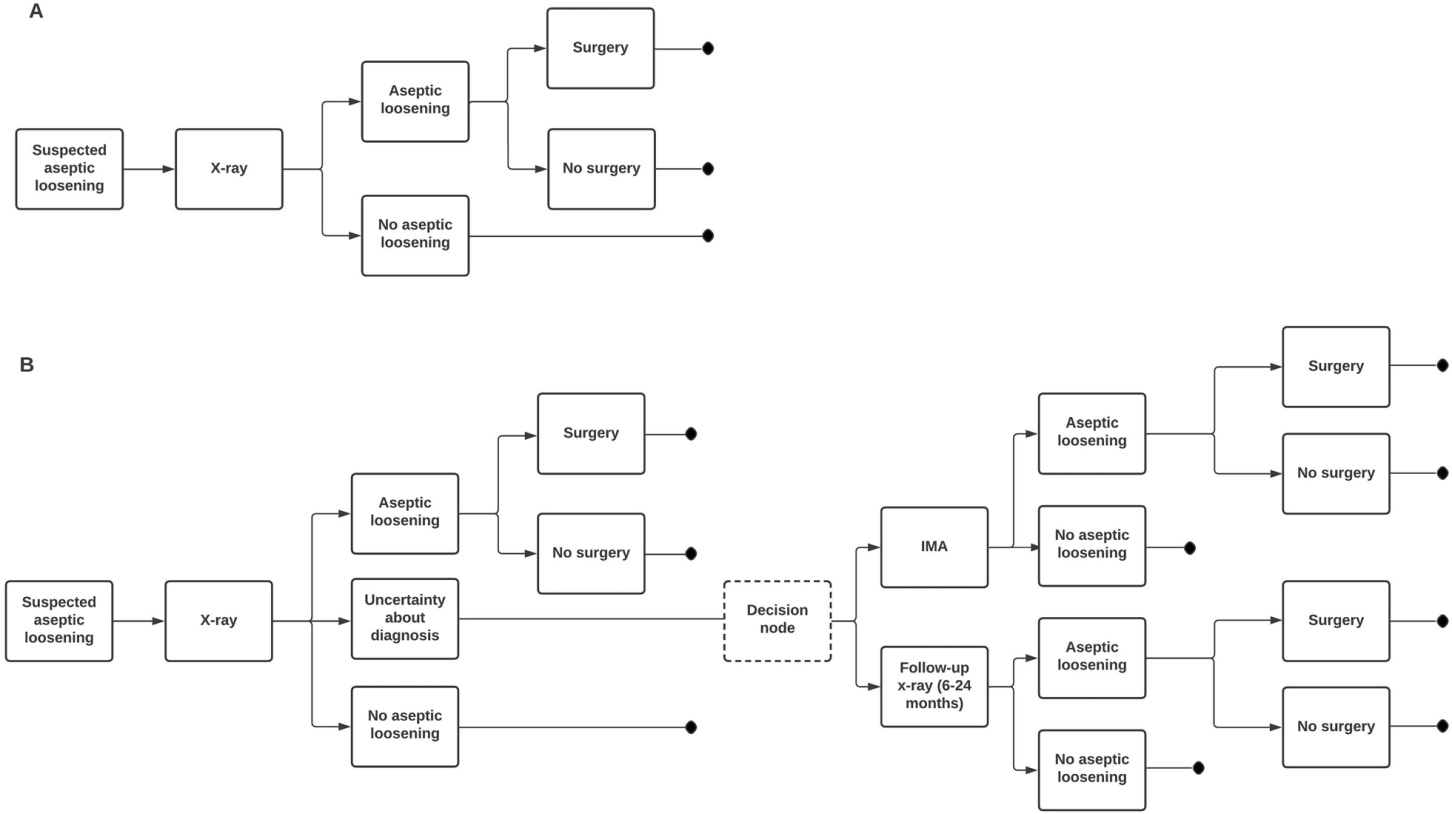
Decision trees depicting the standard diagnostic pathway for patients diagnosed with aseptic loosening. **A** Basic model and **B** IMA diagnostic pathway. **A** The model assumes no uncertainty in the results from an initial X-ray for patients with suspected aseptic loosening. **B** The model includes a group of patients for whom the initial X-ray did not provide conclusive information about aseptic loosening

In the case of aseptic loosening, a hypothetical assumption would be to say that, from the X-ray, an examining physician would immediately know whether the implant is loose. In a basic model (Fig. 1A), there are only two alternative outcomes, or consequences, after an X-ray; two branches indicating aseptic loosening or not (read from left to right). For some additional information to include in the model, it is also possible to allow for patients with diagnosed aseptic loosening, while some patients may choose not to have the recommended surgery.

However, since it is known that the result of an X-ray is not conclusive for all patients, the previous model does not cover all the potential outcomes. There are in fact three alternative outcomes of the X-ray examination: diagnosed as aseptic loosening, no aseptic loosening and an uncertain diagnosis with regard to aseptic loosening. This last group of patients comprises those for whom the clinician would require additional information before making the choice of whether or not to recommend surgery. If the healthcare provider has incorporated IMA in its diagnostic pathway, this could be the natural next step after an inconclusive X-ray examination [18]. If IMA is not available, patients are instead typically referred to at least one follow-up X-ray examination at a later date. The model examined in this study thus includes an additional section (Fig. 1B) in which these patients with an inconclusive initial examination will either be given an additional examination using IMA or be scheduled for a follow-up examination with an X-ray.

### Data collection: probabilities, costs and QALYs

Populating the model includes identifying the probabilities of patients following different paths at each node in the tree and the costs and health outcomes for each branch [17]. In this study, data were collected based on availability from published literature, clinical data and, where no other option was available, from experts’ opinions. The sources of probabilities, then of costs and lastly of health outcomes are listed below.

To identify transition probabilities for the model, opinions from surgeons in the field were used; as sources from literature are either outdated or not specifically related to aseptic loosening [19, 20]. As a result, two different questionnaires were designed. The probabilities of the X-ray path were obtained through a questionnaire sent by email to 14 revision surgeons from different cities in Sweden. A second questionnaire was sent to surgeons with direct experience of IMA or who had followed a cohort of patients on whom IMA was used (see Additional file 1: Appendix S1).

To our knowledge, there are no publications reporting the costs of revision surgery after aseptic loosening. For this reason, cost-per-patient data for healthcare use related to revision surgery in 2018 were obtained for 30 patients with the aseptic loosening of hip implants from the Sahlgrenska University Hospital administrative records. All the costs included in this study are in SEK and recalculating them in EUR (10 SEK ≈ 1 EUR) is straightforward. The costs were divided into different categories: Physician visit, Computed Tomography (CT), X-ray, Follow-up, Surgery, Other treatment and IMA. The cost when surgery could not be planned beforehand (i.e. acute surgery) was also given in the database and used in the sensitivity analysis. The end of each branch accounts for several cost categories, corresponding to all the steps performed in that specific pathway. A physician visit is assumed if a patient undergoes a diagnostic examination. A CT scan is necessary if the physician requests IMA. Other treatment costs are based on painkillers and follow-up visits, based on clinical experience. As a result, the costs for a patient undergoing revision surgery after IMA follow-up include: two physician visits, one X-ray scan, surgery, a CT scan and IMA. In the X-ray/X-ray model, neither CT nor IMA costs are considered. Descriptive statistics were reported for the cost categories. For the surgery cost component, 95% bias-corrected confidence interval were calculated using bootstrap of 1,000 random samples with replacement. Bootstrapping is a statistical resampling method that involves creating multiple datasets by randomly selecting data points with replacement from a single dataset, enabling the estimation of sampling distributions and facilitating a wide range of statistical analyses. The main analysis used mean estimations, while other statistics were used for the sensitivity analyses. The Additional file also gives the descriptive statistics produced for different cost categories (Additional file 1: Table S2).

A literature review was conducted to gather QALYs data for revision surgery after aseptic loosening and alternative treatments. The databases used for the search are Scopus, PubMed and Google Scholar. A timeframe from 2010 to 2022 was set. The search terms were: [(QALYs) OR (Health-Related Quality of Life)] AND (Hip Arthroplasty).

### ICER and threshold

To judge whether IMA is a cost-effective solution for patients undergoing revision surgery, an incremental cost-effectiveness ratio (ICER) will be calculated. It is given by the ratio between the difference in costs between the two models and the corresponding difference in QALYs. This value will be compared with a threshold value indicating society’s willingness to pay for gaining one year of perfect health, i.e. one QALY. Following the NICE guidelines, England and Wales set their threshold at € 35,000 [21]. In Sweden, no official threshold has been released [22]. An informal threshold of ≈ SEK 500,000 is widely used in Sweden. However, it has been reported that the willingness to pay per QALY is higher [23]. In this study, the willingness-to-pay thresholds used to assess the cost-effectiveness of the IMA pathway were both SEK 500,000 and SEK 700,000 per QALY.

### Sensitivity analyses

Findings based on assumptions need to be tested through sensitivity analyses where these can be expected to influence the cost-effectiveness estimate for the intervention under study, in this case the use of IMA for patients with an inconclusive initial X-ray. As ICER is a distribution with uncertainty rather than a single value, a probabilistic sensitivity analysis was performed. A cost-effectiveness plane was built, bootstrapping 1000 repetitions, to help visualize the distribution [24]. In the additional file, transition probabilities were adjusted to incorporate uncertainty in the responses to the physician questionnaires and other model assumptions (Additional file 1: Fig. S1, Table S1). The sensitivity analysis of costs and QALYs tested the assumptions relating to their respective levels in the IMA pathway using descriptive statistics for cost-per-patient data and varying other parameters based on theoretical grounds. For patients receiving surgery immediately, the base case assumed that the HRQoL increases linearly until it reaches a plateau of 0.8 at 18 months (Additional file 1: Fig. S2), while scenario analyses assumed that those patients reach the plateau after just 6 months or not until 24 months. For patients with delayed necessary surgery, a corresponding increase in HRQoL is assumed after their surgery, with the maximum level adjusted for the delay in base care. Scenario analyses assumed a higher increase in HRQoL or a lower increase reaching an even lower final HRQoL. Patients receiving other treatment are assumed to have constant HRQoL, while patients not receiving necessary surgery or other treatment will reduce their HRQoL over time. Additional file 1: Table S3 presents the cost values used in the sensitivity analysis, with reference to each tested assumption. RStudio 2023.09.0–463 was used for the data analysis while Excel 2310 Build 16.0.16924.20054 was used for building the economic model.

### Authorization from Sahlgrenska university hospital

One of the initiators of this project, M.M., is a surgeon at Sahlgrenska University Hospital. The use of IMA in diagnosing aseptic loosening is contested, since it can be claimed that an X-ray provides enough information for an informed decision and the cost–benefit of IMA is unclear. Examining the cost-effectiveness of introducing IMA in different scenarios is thus of interest for the hospital’s development towards the efficient use of resources. Approval for the extraction of cost-per-patient data was given by the head of the orthopedics department at Sahlgrenska University Hospital (see Additional file 1: Appendix S2). The data otherwise used in this study were based on published literature and the anonymous opinions of surgeons and are therefore not covered by ethical approval.

## Results

The online questionnaire for non-IMA surgeons generated 11 responses and the questionnaire for IMA surgeons generated 12 responses. The literature review identified three relevant studies; HRQoL estimates from Konopka et al. [25] were used to estimate the HRQoL improvement after surgery, while estimates from Mota [26] were used for the HRQoL decrease associated with delayed surgery. Estimates from Jönsson et al. [27] were used for patients receiving other treatments (Table 1).

**Table 1:** Transition probabilities, costs, and QALYs used to populate the base case model of IMA vs X-ray in aseptic loosening of hip implants

	Value
Transition probability	
Probability of being loose for aseptic loosening after X-ray	0.7
Probability of being not loose after X-ray	0.15
Probability of uncertain diagnosis after X-ray	0.15
Probability of having revision surgery after diagnosed as loose (X-ray)	0.75
Probability of having another treatment after diagnosed as loose (X-ray)	0.25
Probability of being loose for aseptic loosening after an X-ray (follow-up)	0.3
Probability of being not loose after a second X-ray (follow-up)	0.7
Probability of having revision surgery after diagnosed as loose (X-ray follow-up)	0.75
Probability of having another treatment after diagnosed as loose (X-ray follow-up)	0.25
Probability of being loose after IMA	0.3
Probability of being not loose after IMA	0.7
Probability of having revision surgery after diagnosed as loose (IMA)	0.75
Probability of having another treatment after diagnosed as loose (IMA)	0.25
Costs	
X-ray	SEK 1029
Physician visit	SEK 2635
IMA	SEK 9000
Surgery	SEK 152,187
CT	SEK 4353
Follow-up	SEK 5318
Other treatment	SEK 5318

HRQoL estimates—Baseline value assumed equal for all: 0.62						
Parameters	6 months	12 months	18 months	24 months	QALYs	Reference
Immediate necessary surgery	0.68	0.74	0.8	0.8	1.465	Konopka et al. [25]
Needed surgery (receiving nothing or other treatment)	0.5672	0.5144	0.4616	0.4088	1.0288	Mota [26]
Necessary surgery delayed 1 year	0.5672	0.5144	0.6308	0.7472	1.198	Mota [26]–Konopka et al. [25]
No surgery, receiving other treatment	0.65	0.65	0.65	0.65	1.2925	Jönsson et al. [27]

SEK: Swedish krona, HRQoL: Health-related quality of life, QALYs: Adjusted Quality Life Years. IMA: Implant Movement Analysis, CT: Computed tomography

According to the base case, the mean healthcare cost per patient was SEK 94,184 for the X-ray/IMA pathway, while it was SEK 93,279 for the X-ray/X-ray pathway, thus resulting in incremental costs of SEK 905 for the latter. Each patient in the X-ray/IMA pathway obtained QALYs corresponding to an average of 1.34 years of perfect health during the two-year study period, while the corresponding QALY for the X-ray/X-ray pathway was 1.33, resulting in an incremental effect of 0.009 QALY. The resulting ICER is equal to SEK 99,681 per QALY.

## Sensitivity analysis

**Fig. 2:**
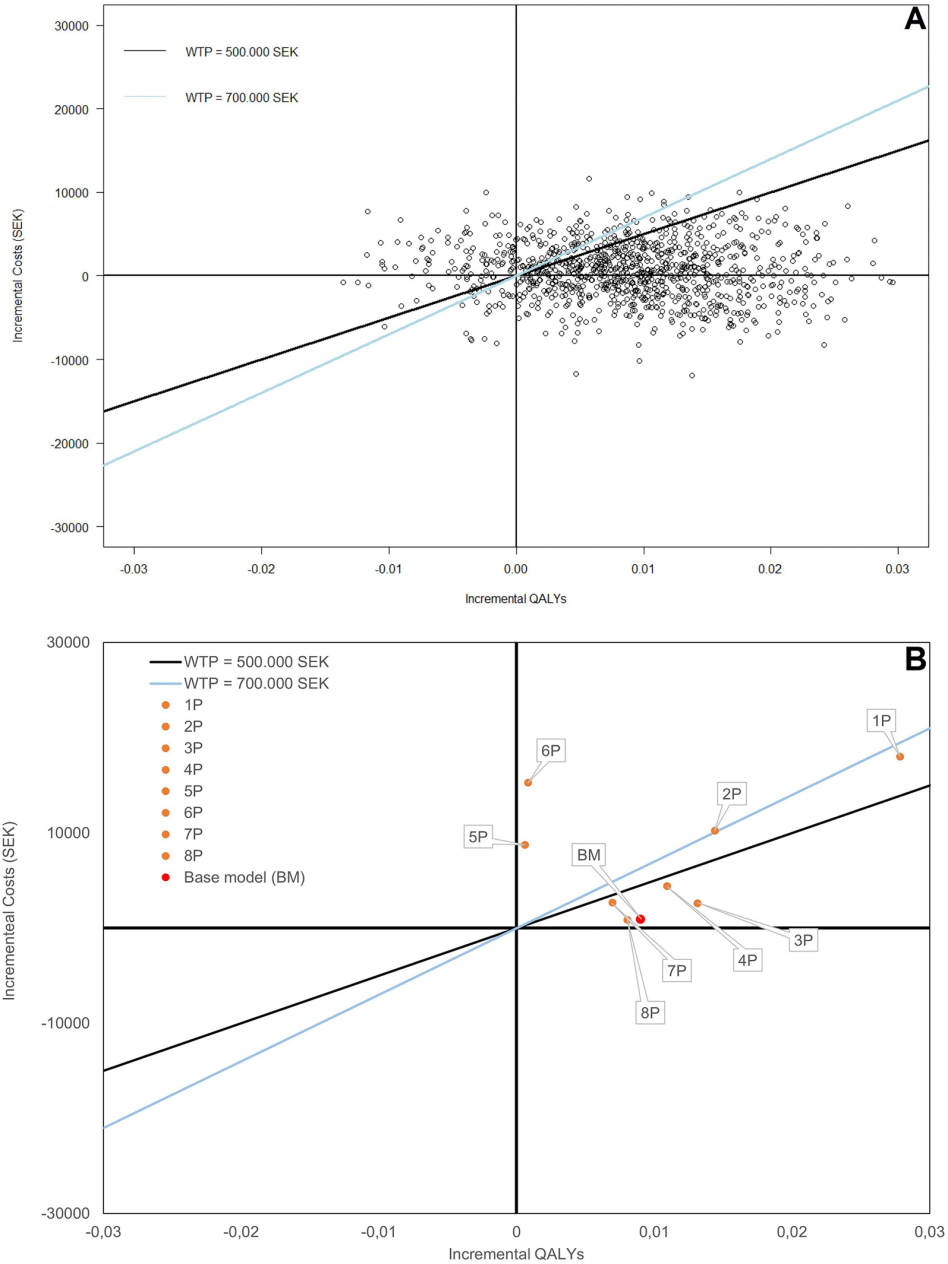
Cost-effectiveness planes produced from; **A** the probabilistic sensitivity analysis and **B** deterministic sensitivity analysis. WTP: Willingness to pay, SEK: Swedish krona, QALYs: Adjusted Quality Life Years, AL: Aseptic loosening, BM: Base model, AM: Alternative model, SEK: Swedish krona

According to the probabilistic sensitivity analysis (Fig. 2A), most of the observations are under both WTPs thresholds. Moreover, the vast majority of observations are localized in the northeast and southeast quadrants, indicating that, as an additional tool, IMA would result in QALY gain.

Furthermore, the sensitivity analysis based on lower and higher estimates for transition probabilities based on the questionnaires (Fig. 2B) found that varying the number of patients that are diagnosed with aseptic loosening at follow-up (3P, 4P, 7P, 8P) resulted in an ICER lower than the SEK 500,000 threshold. However, varying the number that are diagnosed during the initial X-ray (1P and 2P, i.e. clinicians change their behavior based on the knowledge that IMA is available at a later stage of diagnosis) resulted in a higher ICER, albeit still lower than the SEK 700,000 threshold. There are only two scenarios in which the ICERs are over the threshold values. In the 5P scenario, a larger proportion of people will receive immediate surgery, since more patients experience aseptic loosening after the first X-ray and, as a result, the incremental total cost will be higher and the incremental total QALYs will be lower compared with the base scenario. In the 6P scenario, fewer people will receive immediate surgery, but the incremental total cost will be higher and the incremental total QALYs will be lower, compared with the base model.

**Fig. 3:**
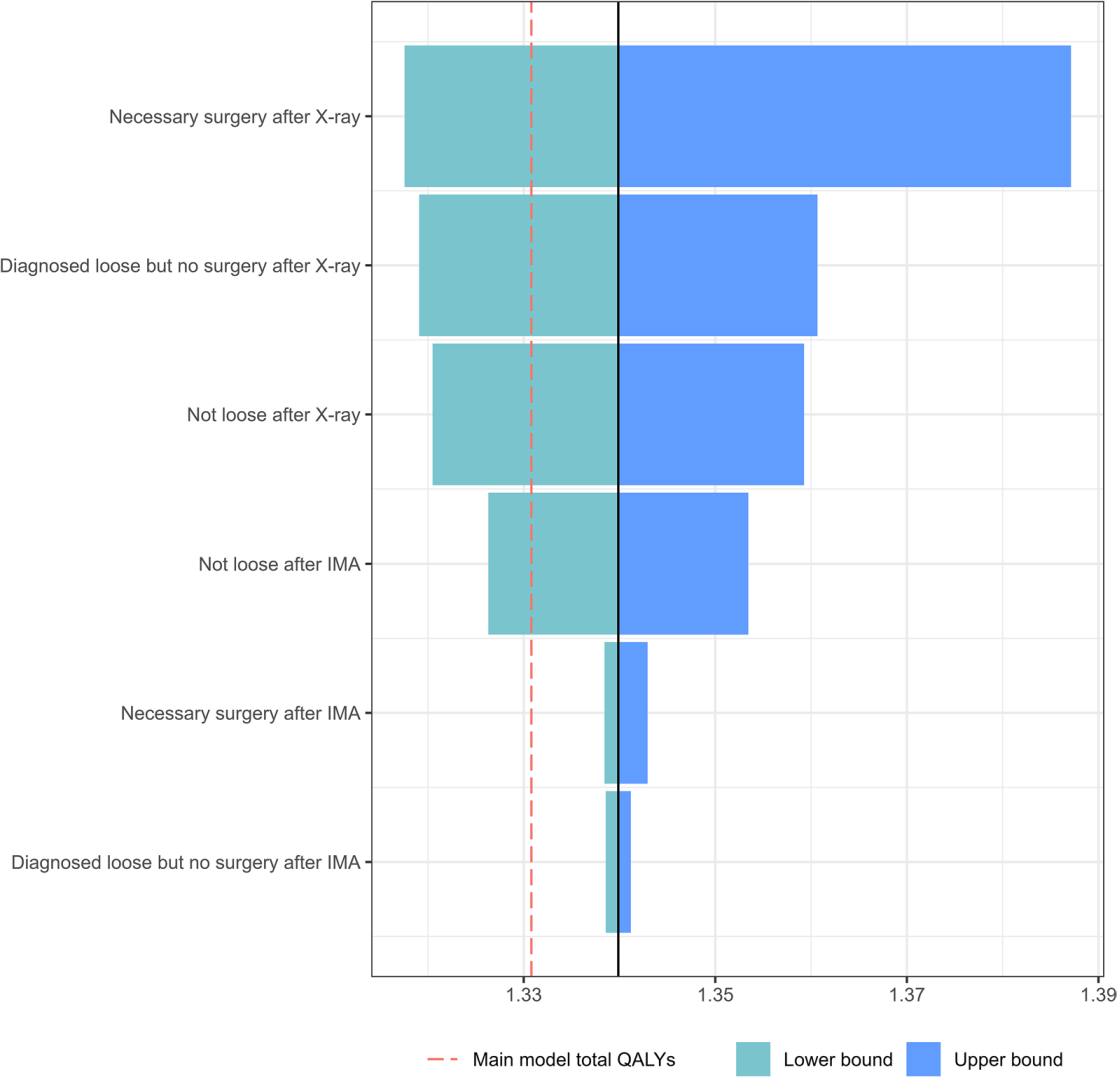
Tornado diagram based on the sensitivity analysis made on the QALYs for the IMA pathway. IMA: Implant Movement Analysis, QALYs: Quality Adjusted Life Years

According to the tornado diagram for QALYs (Fig. 3), the QALY used for surgery directly after the first X-ray and for individuals with aseptic loosening who decided not to accept surgery were the main drivers of variation in the QALY estimates for the X-ray/IMA pathway.

**Fig. 4:**
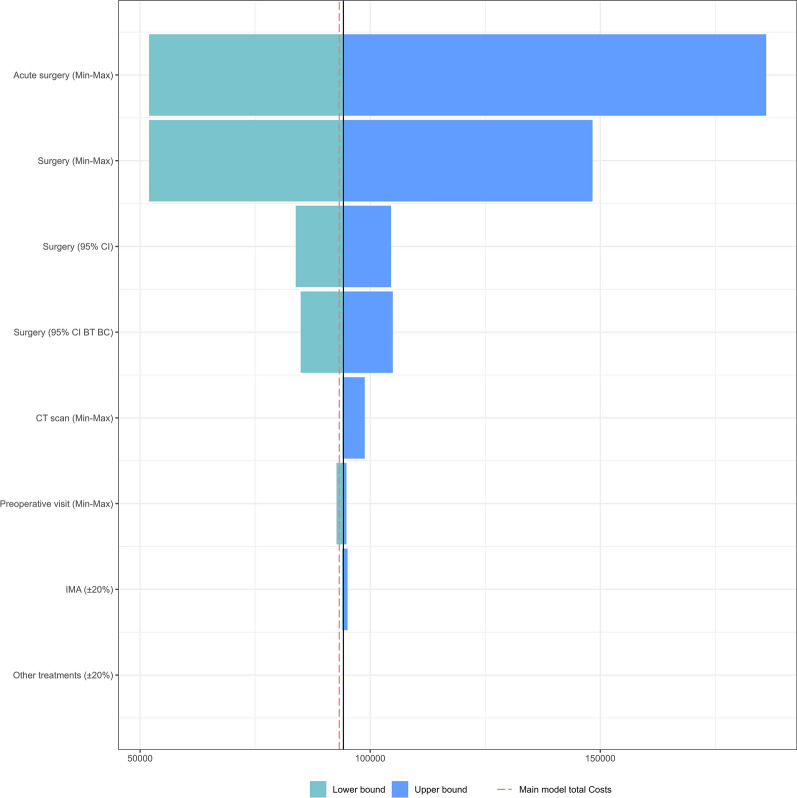
Tornado diagram based on the sensitivity analysis made on the costs for the IMA pathway. CT: Computed Tomography, IMA: Implant Movement Analysis, BT: Bootstrapped, BC: Bias-corrected, Min: Minimum, Max: Maximum, CI: Confidence Interval

According to the tornado diagram of costs (Fig. 4), surgery costs caused the largest variation in per-patient costs for the X-ray/IMA pathway.

## Discussion

According to this health-economic model, IMA is a cost-effective tool for diagnosing aseptic loosening among patients with inconclusive initial X-ray examinations, mainly because it provides a faster diagnosis compared with a follow-up X-ray after several months. In the base case, the ICER was SEK 99,681 per QALY, which is far lower than the SEK 500,000 used as an informal WTP threshold in Sweden. In the sensitivity analyses, the X-ray/IMA pathway remained cost effective except when changing the underlying assumption that only patients with an inconclusive X-ray were sent for IMA. Furthermore, changing QALYs and costs based on uncertainty found that IMA costs and downstream factors had little impact on the results, while estimates for the initial surgery had a greater impact. The finding thus emphasizes the importance of the initial diagnosis not being misleading or misinterpreted. This would result in both more costs for the healthcare system and delayed recovery for patients.

Based on the literature, it appears that the overall price of a revision surgery is higher, sometimes reaching two or three times the price identified for aseptic loosening surgery [28–31]. Cost levels are often difficult to translate between countries and, as a result, future studies should be conducted to assess this difference in one population, since different surgery costs accounted for the largest variation in total costs, while undergoing a necessary surgery after the first X-ray, being diagnosed with aseptic loosening but not having a surgery after the first X-ray and not having aseptic loosening after the first X-ray accounted for the largest variation in the total QALYs. This underlines how crucial it is to choose carefully when it comes to performing revision surgery and when not to, given the burden of the cost on the healthcare system. Furthermore, the first X-ray scan accounts for a large variation in QALYs gained, implying that misdiagnosis can greatly reduce patients’ quality of life.

### Limitations

The main limitation of the study was that a great deal of empirical data was lacking and had to be substituted with expert opinion. While transition probabilities were gathered from more than 30 surgeons with and without IMA experience around Sweden, their responses showed large variation. This could potentially indicate a difference in the underlying patient population. While a larger sample of surgeons might have improved the data, the novelty of IMA would have resulted in most additional surgeons in the group having no previous experience of IMA and the data on IMA use not being improved to any great extent.

For this study, a small sample of cost-per-patient data was obtained to indicate costs and, while a larger sample might have provided more information, this choice was based on the expectation that costs are similar between patients in this population due to the grouping that is performed at the hospital into diagnosis-related groups, which in turn forms the basis of funding to the ward. Additionally, no indirect costs were assessed, which is a limitation and would otherwise provide a more accurate estimation of the total impact of introducing a diagnostic tool like IMA to society. It is expected that adding indirect costs would benefit the X-ray/IMA pathway, since increased QALYs have the potential to result in increased availability for productive work. However, many people in the target population have retired and are thus exempt from most methods for measuring indirect costs.

The use of QALYs in our study is crucial for a holistic assessment of hip replacement’s impact on both quality of life and survival, as well as for supporting cost-effectiveness analyses and enhancing patient-centered outcomes.

From a review of the literature, research on hip replacements is often limited to the initial revision and potentially the probabilities of experiencing aseptic loosening, but it lacks revision outcomes. As a result, previous studies of QALY after revision surgery for aseptic loosening are fairly scarce.While some assumptions had to be made, it appears from the sensitivity analyses that the assumptions related to the follow-up after an initial inconclusive X-ray had less influence on our results.

Lastly, the difficulties involved in generalizing our study to the general population with aseptic loosening are evident, since not many people currently use IMA as a diagnostic tool, resulting in data scarcity. Conclusively, this modeling study provides an initial estimate of the cost-effectiveness of IMA in diagnosing aseptic loosening. However, it is crucial to acknowledge that the development of a more advanced model is hindered by the scarcity of empirical data. Therefore, further research is essential to refine the model and address the limitations imposed by data constraints.

## Conclusions

A diagnostic pathway using IMA after an inconclusive X-ray for suspected aseptic loosening was cost effective compared with a pathway with X-ray follow-up alone.

### Supplementary Information

**Additional file 1:** **Figure S1.** Decision tree showing where transition probabilities were altered and analysis performed. **Figure S2.** Sensitivity analysis made on different Health-Related Quality of Life categories. **Table S1.** Values used in the sensitivity analysis of alternative transition probabilities. **Table S2.** Descriptive costs statistics from the hospital Cost Per Patient data. **Table S3.** Values used in the sensitivity analysis of alternative cost and QALY levels. **Appendix S1.** Summary of questionnaire responses. **Appendix S2.** Authorization from Sahlgrenska University Hospital

## Data Availability

Not applicable.
