# Supplementary material for: Cost-effectiveness of implant movement analysis in aseptic loosening after hip replacement: a health-economic model
Source: Cost Eff Resour Alloc. 2023 Nov 20;21:88. doi: 10.1186/s12962-023-00498-w (PMC10662297; doi:10.1186/s12962-023-00498-w)
Supplement: Supplementary file 1 — Additional file 1: Figure S1. Decision tree showing where transition probabilities were altered and analysis performed. Figure S2. Sensitivity analysis made on different Health-Related Quality of Life categories. Table S1. Values used in the sensitivity analysis of alternative transition probabilities. Table S2. Descriptive costs statistics from the hospital Cost Per Patient data. Table S3. Values used in the sensitivity analysis of alternative cost and QALY levels. Appendix S1. Summary of questionnaire responses. Appendix S2. Authorization from Sahlgrenska University Hospital [file 12962_2023_498_MOESM1_ESM.pdf]

Supplemental material

Cost -effectiveness of Implant Movement Analysis in aseptic loosening after hip replacement: a health economic model

Authors:

Davide Lovera[1,2]

Olof Sandberg[2]

Maziar Mohaddes[3]

Hanna Gyllensten[4]

Affiliations

[1] Sahlgrenska Academy, University of Gothenburg, Gothenburg, Sweden.

[2] Sectra, Linköping, Sweden.

[3] Department of Orthopaedics, Institute of Clinical Sciences, Sahlgrenska Academy, University of Gothenburg

[4] Institute of Health and Care Sciences, Sahlgrenska Academy, University of Gothenburg, Gothenburg, Sweden.

Corresponding author

Hanna Gyllensten, Institute of Health and Care Sciences, Sahlgrenska Academy, University of Gothenburg, Postal Address: Box 457, SE-405 30 Gothenburg, Sweden, E-mail: hanna.gyllensten@gu.se. Phone: +46-(0)70-748 24 12

Content

Figure S1: Decision tree showing where transition probabilities were altered and analysis performed…………………………………………………………………..……………….3

Table S1: Values used in the sensitivity analysis of alternative transition probabilities...........4

Table S2: Descriptive costs statistics from the hospital Cost Per Patient data………………..5

Figure S2: Sensitivity analysis made on different Health-Related Quality of Life categories..6

Table S3: Values used in the sensitivity analysis of alternative cost and QALY levels...7

Appendix S1: Summary of questionnaire responses.……………………………………....8

Appendix S2: Authorization from Sahlgrenska University Hospital………………………..10

Figure S1: Decision tree showing where transition probabilities were altered and analysis performed.

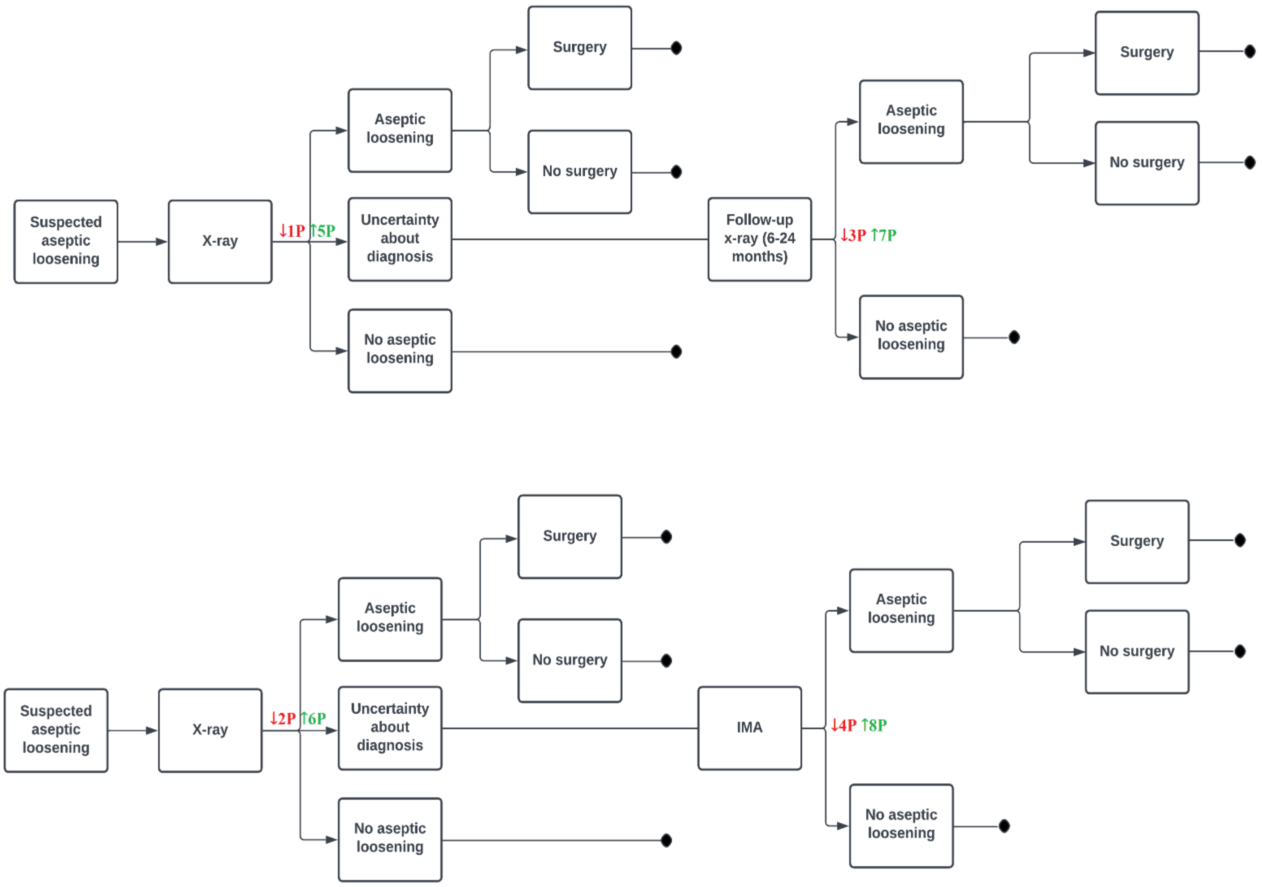

Abbreviations: *IMA*, Implemented Movement Analysis**.**

Table S1: Values used in the sensitivity analysis of alternative transition probabilities.

Decision tree branch**	Branch	No.	Transition probability	Main	No.	Transition probability
**Change to X-ray/X-ray pathway:** fewer (1P) / more (5P) patients diagnosed with aseptic loosening at initial X-ray. Thus, the difference indicate that clinicians chose to send more/less patients to follow-up X-ray.	Aseptic loosening after first x-ray	1P	0.50	0.70	5P	0.80
	Uncertain after first x-ray		0.30	0.15		0.10
	No loosening after first x-ray		0.20	0.15		0.10
**Change to X-ray/IMA pathway** fewer (2P) / more (6P) patients are sent to IMA due to uncertain initial X-ray. Thus, the difference indicate that clinicians chose to send more/less patients to follow-up IMA.	Aseptic loosening after first x-ray	2P	0.80	0.70	6P	0.50
	Uncertain after first x-ray		0.10	0.15		0.30
	No loosening after first x-ray		0.10	0.15		0.20
**Change to X-ray/X-ray pathway:** more (3P) / fewer (7P) patients diagnosed with aseptic loosening at follow-up X-ray. Thus, the difference indicate that more/less of uncertain cases are actually loose and can be found at follow-up X-ray.	Aseptic loosening after second x-ray	3P	0.50	0.30	7P	0.20
	No loosening after second x-ray		0.50	0.70		0.80
**Change to X-ray/IMA pathway:** more (4P) / fewer (8P) patients diagnosed with aseptic loosening at follow-up IMA. Thus, the difference indicate that more/less of uncertain cases are actually loose and can be found at follow-up IMA.	Aseptic loosening after second x-ray	4P	0.50	0.30	8P	0.20
	No loosening after second x-ray		0.50	0.70		0.80

* X-ray/X-ray is the standard pathway patients with aseptic loosening, with X-ray follow up used after uncertain initial X-ray examination; X-ray/IMA is the new pathway where IMA is available for the follow up.

Abbreviations: *ICER*, Incremental cost-effectiveness ratio; *IMA*, Implemented Movement Analysis.

Table S2: Descriptive costs statistics from the hospital Cost Per Patient data.

	Surgery cost	Acute surgery	Physician visits	CT	X-ray	Follow-up (all costs after surgery)
	n=27	n=3	n=31	n=12	n=10	n=9
	SEK (No.)	SEK (No.)	SEK (No.)	SEK (No.)	SEK (No.)	SEK (No.)
**Mean**	152,187	28,0355	2,635	4,353	1,029	5,318
**Standard Deviation**	49,104	38,532	323	1,115	294	3,543
**Median**	138,747	284,660	2,752	3,802	1,091	5,666
**Minimum**	76,640 (1C)	239,852 (7C)	1,623 (9C)	3,802 (11C)	545	1,396
**Maximum**	249,035 (2C)	316,554 (8C)	2,888 (10C)	7,584 (12C)	1,562	12,687
**Mean-CI95**	133,666 (3C)	N/A	N/A	N/A	N/A	N/A
**Mean+CI95**	170,709 (4C)	N/A	N/A	N/A	N/A	N/A
**Mean-CI95 BT BC**	135,581 (5C)	N/A	N/A	N/A	N/A	N/A
**Mean+CI95 BT BC**	171,377 (6C)	N/A	N/A	N/A	N/A	N/A

BC: Bias corrected, BT: Bootstrapped, CI: Confidence Interval.

Figure S2: Sensitivity analysis made on different Health-Related Quality of Life categories.

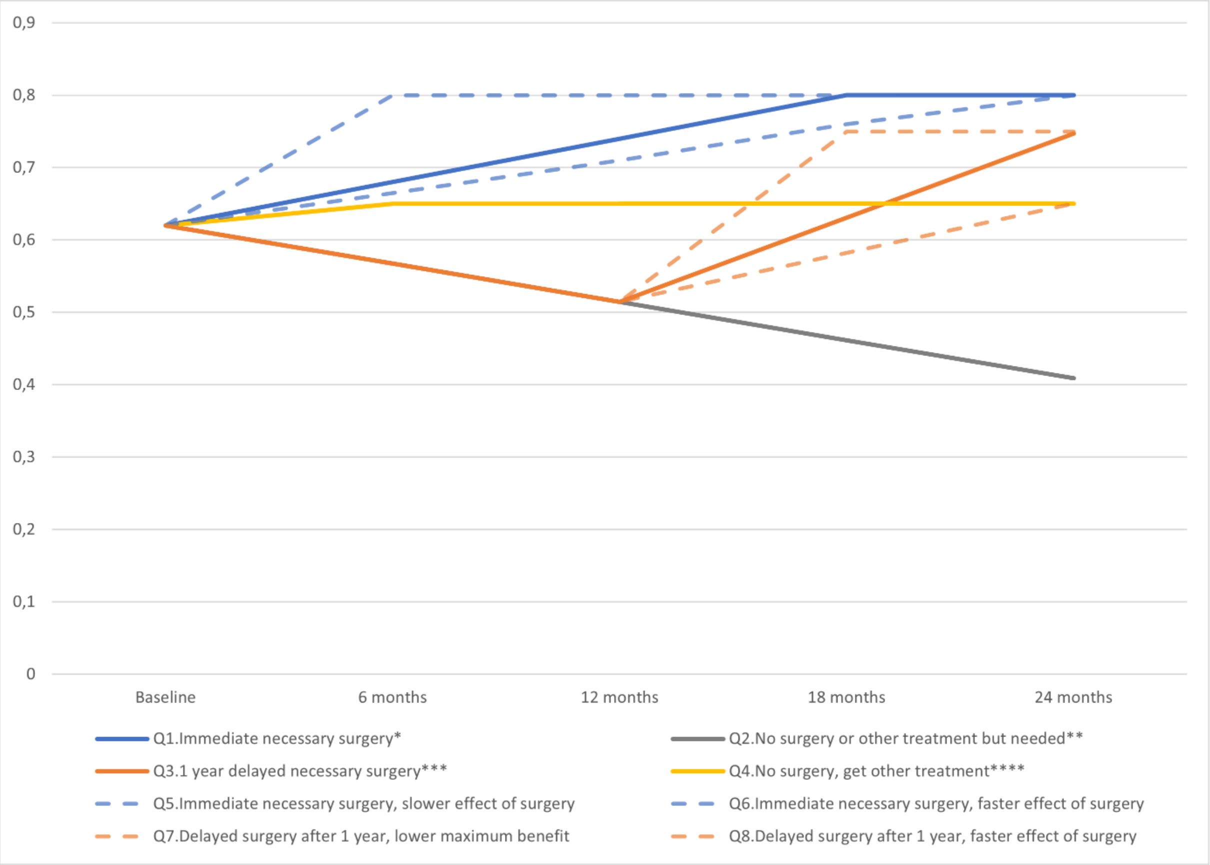

Table S3: Values used in the sensitivity analysis of alternative cost and QALY levels.

**Costs**	**Unit (SEK)**	**Total (SEK)**
**Base model**		
Standard pathway	N/A	93,279
IMA pathway	N/A	94,184
**Sensitivity analysis (Source)**		
Minimum *surgery* cost (Table S2: 1C)	76,640	51,953
Maximum *surgery* cost (Table S2: 2C)	249,035	148,322
Lower 95% CI limit *surgery* cost (Table S2: 3C)	133,666	83,830
Upper 95% CI limit *surgery* cost (Table S2: 4C)	170,709	104,537
Lower BT/BC 95% CI *surgery* cost (Table S2: 5C)	135,581	84,901
Upper BT/BC 95% CI *surgery* cost (Table S2: 6C)	171,377	104,911
Minimum acute surgery cost used for all *surgery* (Table S2: 7C)	239,852	143,188
Maximum acute surgery cost used for all *surgery* (Table S2: 8C)	316,554	186,065
Lower cost for *IMA*, compared to the base case, estimated	7,000	93,884
Higher cost for *IMA*, compared to the base case, estimated	15,000	95,084
Lower cost for *physician visits* (Table S2:9C)	1,623	92,685
Higher cost for *physician visits* (Table S2:10C)	2,888	94,847
Lower cost for *other treatment*, compared to base case, -20%	4,290	94,172
Higher cost for *other treatment*, compared to base case, +20%	6,346	94,195
Lower cost for *CT*, compared to the base case (Table S2:11C)	3,802	93,979
Higher cost for *CT*, compared to the base case (Table S2:12C)	7,584	98,811
**Health outcomes**	**Unit (QALY)**	**Total (QALY)**
**Base model**		
Standard pathway	N/A	1.331
IMA pathway	N/A	1.340
**Sensitivity analysis**		
Lower QALY improvement from *necessary surgery after initial X-ray* (Figure S2:Q5)	1.423	1.318
Higher QALY improvement from *necessary surgery after initial X-ray* (Figure S2:Q6)	1.555	1.387
Lower QALY-estimate if no surgery after X-ray even if AL	1.148	1.319
Higher QALY-estimate if no surgery after X-ray even if AL	0.910	1.361
Lower QALY improvement from n*ecessary surgery after IMA* (Figure S2:Q5)	1.423	1.338
Higher QALY improvement *from necessary surgery after IMA* (Figure S2:Q6)	1.555	1.343
Lower QALY-estimate if no surgery after IMA even if AL	1.148	1.339
Higher QALYs-estimate if no surgery after IMA even if AL	0.910	1.341
Lower QALY improvement if not loose after IMA	1.422	1.326
Higher QALY improvement if not loose after IMA	1.163	1.353
Lower QALY improvement if not loose after X-ray	1.422	1.321
Higher QALY improvement if not loose after X-ray	1.163	1.359

Figures are rounded.

AL: Aseptic loosening, BT: Bootstrapped, BC: Bias corrected, CPP cost per patient, CT: Computed Tomography, IMA: Impact movement analysis, QALYs: Quality-adjusted life years, SA: Sensitivity analysis, SEK: Swedish Krona, N/A: Not Available.

Appendix S1: Summary of questionnaire responses.

Questionnaire sent out to non-IMA surgeons

**1.**	The X-ray scan indicates aseptic loosening and I would recommend surgery
**2.**	The X-ray scan does not indicate aseptic loosening
**3.**	I am uncertain – the available information does not give conclusive information about recommending surgery or not and this patient will be scheduled to come back for a new assessment later.
**4.**	Among the cases classified as **loose after an X-ray** (**statement 1 above**), how many out of 100 such cases get revision surgery within 2 years?
**5.**	Among the cases classified as **not loose** (**statement 2 above**), how many out of 100 such cases will still get revision within 2 years?
**6.**	Among the cases classified as **uncertain cases** (**statement 3 above**), how many out of 100 such cases will get a revision within 2 years?
**7.**	How often, during a surgery of aseptic loosening, do you realize that the implant was not as loose as initially assessed from the x-ray? Consider 100 surgeries as an example.
**8.**	If you have any additional comments, please provide them below.

Questionnaire sent out to IMA surgeons

**1.**	The X-ray scan indicates aseptic loosening and I ***would recommend surgery***
**2.**	The X-ray scan ***does not indicate*** aseptic loosening
**3.**	***I am uncertain*** – the available information does not give conclusive information about recommending surgery or not and this patient will be sent on an IMA
**4.**	Among the cases classified as **loose after an X-ray** (**statement 1 above**), how many out of 100 such cases get revision surgery within 2 years?
**5.**	Among the cases classified as **not loose** (**statement 2 above**), how many out of 100 such cases will still get revision within 2 years?
**6.**	Among the cases sent for IMA (**statement 3 above**) how many out of 100 such cases will get a revision within 2 years?
**7.**	Among the cases sent for IMA (**statement 3 above**) **and not receiving revision afterwards**, how many out of 100 of these cases will still get a revision within 2 years (due to changed circumstances/information)?
**8.**	How often, during a surgery of aseptic loosening where IMA has been used, do you realize that the implant was not as loose as initially assessed from the IMA? Consider 100 surgeries as an example.
**9.**	If you have any additional comments, please provide them below.

Descriptive statistics for the answers in the non-IMA group

Variable	Obs	Mean	SD	Q1	Median	Q3	Min	Max
2a1	12	57.50	26.67	35	70	80	10	90
2a2	12	41.67	33.80	15	25	65	10	100
2a3	12	28.33	23.29	10	20	40	10	80
2b1	0	.	.	.	.	.	.	.
3a1	12	68.33	22.90	55	75	80	30	100
3b1	12	22.50	10.55	20	20	25	10	50
3b2	12	17.50	13.57	10	10	20	10	50
4	12	19.17	15.64	10	10	20	10	60

Obs: Observations, SD: Standard Deviation, Q1: First Quartile, Q3: Third Quartile, Min: Minimum value, Max: Maximum value, 2a1: Aseptic loosening and surgery is recommended, 2a2: Uncertain,2a3: No aseptic loosening, 2b1: Surgery after IMA, 3a: Immediate surgery, 3b1/2: Delayed surgeryafter uncertain or not loose, respectively, 4: Unnecessary surgery

Descriptive statistics for the answers in the non-IMA group

Variable	Obs	Mean	SD	Q1	Median	Q3	Min	Max
2a1	11	68.18	19.40	50	70	80	30	100
2a2	11	40.91	35.06	10	30	70	10	100
2a3	11	21.82	12.50	10	20	30	10	50
2b1	11	52.73	27.60	30	40	90	20	100
3a1	11	78.18	16.01	70	80	90	40	90
3b1	11	30.00	24.90	10	20	40	10	90
3b2	11	19.09	9.44	10	20	30	10	30
4	11	14.55	6.88	10	10	20	10	30

Obs: Observations, SD: Standard Deviation, Q1: First Quartile, Q3: Third Quartile, Min: Minimum value, Max: Maximum value, 2a1: Aseptic loosening and surgery is recommended, 2a2: Uncertain, 2a3: No aseptic loosening, 2b1: Surgery after IMA, 3a: Immediate surgery, 3b1/2: Delayed surgery after uncertain or not loose, respectively, 4: Unnecessary surgery

Appendix S2: Authorization from Sahlgrenska University Hospital.

Sahlgrenska Universitetssjukhuset

Uppdragstilldelning – tilldelning av särskilda uppdrag

Uppdragstagarens namn:	Befattning/funktion: (anställd som, studerande inom, projektanställd, m.m.)
Maziar Mohaddes<br>**Eventuellt VGR-ID:** mazmo1	Docent, Överläkare
Handledare: (om student)	Verksamhet:
Klicka här för att ange text.	Klicka här för att ange text.

Uppdrag

Kortfattad beskrivning av kvalitetsutvecklingsuppdraget:	
Sammanställa kostnader för patienter opererade med aseptisk höftprotes lossning på Sahlgrenska universitetssjukhuset. Jämförelse av reell kostnad mot hur kliniken ersätts. Utvärdering av ifall merkostnader för en ny preoperativ undersökningsmetod kan motiveras	
Begränsning/särskilda förbehåll:	
Klicka här för att ange text.	
Tidsperiod:	
**fr.o.m:** 2020-03-24	**t.o.m:** 2020-12-31

Underskrift verksamhetschef eller enhetschef efter delegation från verksamhetschef

24/3-20

Datum och namnteckning

Underskrift av uppdragstagare

2020-03-24

Datum och namnteckning

Bakgrund

I Sahlgrenska Universitetssjukhusets "Riktlinje för åtkomst till patientuppgifter" finns beskrivet hur patientdatalagens ska tillämpas. När du deltar i vården av en patient får du, när du tilldelats behörighet till journalsystem eller andra system (såväl analoga som digitala) innehållande patientuppgifter, ta del av patientuppgifter enligt patientdatalagens regler. Detsamma gäller när du enligt din arbetsbeskrivning har rätt till att ta del av patientuppgifter för olika ändamål i hälso- och sjukvården, till exempel administration.

Utöver möjligheten att ta del av patientuppgifter enligt ovan har verksamheten behov av kvalitetsuppföljning där åtkomst till patientdata är nödvändigt. Exempel på uppdrag kan vara:

- Ta del av uppgifter från verksamheten i syfte att systematiskt utvärdera kvalitet och resultat inom
- verksamheten.
- Ta fram statistik.
- Ta fram kliniska fallbeskrivningar till undervisning.
- Ta fram kliniska fallbeskrivningar till kollegiala kvalitetsdiskussioner.

Tänk på att:

- Redovisning av kvalitetsuppdraget ska ske endast på gruppnivå, utan möjlighet till identifiering av enskilda patientuppgifter.
- Det inte är tillåtet att spara patientuppgifter i någon form.
- Arbetsmaterial som t.ex. minnesanteckningar och utkast till journalanteckningar inte får innehålla uppgifter som kan möjliggöra för utomstående att identifiera patienter.
- Arbetsmaterial ska förstöras efter att arbetsuppgiften slutförts.
- Sedvanliga sekretessregler gäller. Sekretessen och tystnadsplikten gäller även efter avslutat uppdrag.

Blankett diarieförs inom verksamheten, uppdragstagare erhåller kopia.
Loggranskning sker enligt gällande rutin.

www.sahlgrenska.se

VÄSTRA GÖTALANDSREGIONEN
SAHLGRENSKA UNIVERSITETSSJUKHUSET

Sahlgrenska Universitetssjukhuset
Kansli och juridik
